# The Statewide Campus System Scholarly Activity Developmental Planning Framework for Community-Based GME Leaders

**DOI:** 10.51894/001c.6521

**Published:** 2018-04-27

**Authors:** William Corser, Brandy Church, Jonathan Rohrer, Kari Hortos

**Affiliations:** 1 Michigan State University Statewide Campus System, College of Osteopathic Medicine, East Lansing, MI 48824

**Keywords:** community-based, graduate medical education, scholarly activity

## Abstract

**CONTEXT:**

During recent years, Graduate Medical Education (GME) leaders in the United States of America have witnessed many substantive changes, including movement to a single accreditation system under the Accreditation Council for Graduate Medical Education. Both MD- and DO-trained residents and faculty must now meet an increasingly stringent set of accreditation standards outlined in Next Accreditation System standards. Specifically, updated scholarly activity standards emphasize a consistent volume and quantity of quality improvement/research projects and dissemination products. The GME literature to date has frequently provided general commentaries regarding individual project strategies or oriented to settings with greater project-related resources. There have also been few articles offering scholarly activity planning strategies for community-based GME officials striving to increase scholarly activity levels.

**PROPOSED PLANNING FRAMEWORK:**

The authors propose a customizable assessment-planning framework, largely derived from their combined decades of consultation experiences with hundreds of community-based resident and faculty projects. The authors will first describe the primary elements of their proposed scholarly activity planning approach for GME leaders so often subject to worsening resource constraints. They will describe six ongoing developmental strategies with several exemplars described. Such a framework will likely require ongoing reassessments and modification.

**CONCLUSIONS:**

The authors hope that this proposed planning framework will offer GME administrators, faculty and residents with a pragmatic set of strategies to develop scholarly activity projects and supports. Ideally, GME leaders can use this approach to inform their design of a sustainable system-customized infrastructure of scholarly activity supports.

## INTRODUCTION

During recent years, the nation’s graduate medical education (GME) leaders in the United States have witnessed substantive changes, including movement to a single accreditation system under the Accreditation Council for Graduate Medical Education (ACGME). Both MD and DO-trained resident and faculty physicians must now meet an increasingly stringent set of accreditation standards outlined in *Next Accreditation System* (NAS) standards.^1^

One particular standard that many GME authors have struggled to address relates to the enhanced requirements for *scholarly activity* (SA) projects.^2–8^ These types of research or quality improvement/patient safety (QIPS) projects are generally conducted to improve GME educational processes, patient care outcomes, provider effectiveness, etc.^2,5,7^ In this paper, the authors will use the abbreviation *SA* to refer to both research and systems-oriented QIPS designs. Although both academic and community-based GME physicians contend with increasing resource constraints, a growing number of authors have contended that these types of challenges may be greater (or certainly different) in community-based GME settings.^9–14^

Unfortunately, GME officials still lack a uniform definition of what comprises a minimal level of SA.^3,8^ Still, the ACGME has released two initial files to guide GME leaders. The first was a 2009 non-validated metrics point system for GME officials and accreditation reviewers gauging resident and faculty SA productivity levels.^15^ Second, a specialty-specific set of SA measurement parameters for residents and fellows was last updated in later 2017.^16^ However, ACGME expectations for SA continue to vary significantly across clinical specialty groups.^4,5,12^

More GME experts have concluded that community-based physicians may typically experience greater difficulty planning and conducting SA projects due to fewer available resources (e.g., library staff, data-capable personnel, analytic experts).^12,17–20^ Most of the SA literature to date appears to be aggregated primarily within two categories. The first, consists of fairly general commentaries.^1–3,11,21,22^ The second is restricted to academic settings concluded to possess greater project resources.^8,22–32^ A growing number of GME authors have called for pragmatic planning strategies geared for community-based leaders striving to address increased SA expectations.^4,6,8,20,25–27,32–34^ In response, the purpose of this paper will be to propose a detailed and customizable planning framework for GME leaders who are working to increase the level of SA at their community-based hospitals.

Some GME authors have implemented SA resident teams, generally comprised of residents from multiple programs with complementary SA project interests.^21,22,31^ Annual residency sequences have been used to equip earlier-year residents with project skills needed to eventually lead their own SA projects.^17,22,31,32,34–36^ Similar to what the Michigan State University Statewide Campus System (SCS) ^37^ authors have seen in affiliated systems, SA councils/committees can also coordinate individual resident and faculty-resident projects.^14,38–40^

GME administrators have appointed later-year residents to chief research/QIPS resident positions to coordinate SA projects in some settings.^36,38,40–42^ Protected time resident rotations with QIPS department personnel may help them generate ideas for later SA projects.^8,18,43–50^ Somewhat similar to the ACGME points system,^15^ some residency program officials have created customized scoring systems to track residents’ SA productivity.^8,51–53^

Many authors have argued that any such mechanisms need to be modified due to system or program-specific influences, and that a standardized rollout approach is unlikely to work across diverse GME settings.^10,37,39,54,55^ For example, the SCS authors and others have described overt discomfort/resistance from some faculty expected to mentor residents during SA project design and conduction activities.^11,13,39,49,50,54–57^ In fact, more recently-trained residents may be more functionally prepared to conduct SA projects than their clinically-experienced faculty.^10,11,14,30,34,41^ Other setting constraints may include inadequate time,^4,6,10,25,27,34,38,39,41^ or uncertainty concerning readily available resources.^4,9,22,30,53^

Numerous experts have already shown that customized SA planning tools (e.g., systematic needs assessment forms, planning templates, annual resident checklists and timelines, etc.) can help facilitate SA project development.^6,10,19,30,58–63^ However, the authors of this paper will propose that a more comprehensive set of customizable SA strategies is generally required to augment system-specific project resources over time.

The SCS authors have developed this comprehensive planning approach from their 2016-2017 experiences consulting on over 210 (i.e., over 1,670 consultation hours to date, mean of 7.95 hours per project (range from 1 to 52 hrs.) community-based SA projects. Resident and faculty project leaders have requested SA consultations from many of the 190+ SCS-affiliated residency programs based at 37 healthcare systems. This paper will: a) describe the key elements of the authors’ suggested SA developmental planning approach, and b) discuss six **non-sequential and ongoing** strategies to enable community-based GME leaders to facilitate increased SA levels. The authors will also describe several exemplary settings with ongoing SA infrastructure developments.

## SCHOLARLY ACTIVITY DEVELOPMENTAL FRAMEWORK

The first goal of this proposed SA planning framework is to provide community-based GME leaders with a specific set of criteria to assess their current in-house project resources and complexities. The authors propose that this approach will prove especially important for GME officials striving to first develop or improve their SA project supports. The authors have repeatedly seen that many community-based leaders’ success in increasing their SA levels have required a broader perspective than simply completing a series of individual projects and dissemination products. In this paper, they assert that the longer-term development of a sustainable SA infrastructure of project supports will prove integral for maintaining SA productivity in lesser-resourced GME settings.

The key elements of this planning framework (see Figure 1) are largely derived from the authors’ successful workshop, online module and individual consultation experiences with community-based GME leaders. They have concluded that many faculty and residents develop idealized (i.e., often unfeasible) projects without capitalizing on on-site resources, and/or conduct projects without the *lessons learned* from earlier SA projects in their healthcare system. GME experts have increasingly supported this broader type of ongoing self-learning model of SA development that entails ongoing reassessments and modification efforts.^4,8,10,30,33,64^ GME authors have also concluded that a *stop and start* approach to SA will impede the development of a sustainable SA project support infrastructure so later project leaders may feel as if they are *starting from scratch*.^5,6,10,12,24,38,42^

**Figure:**
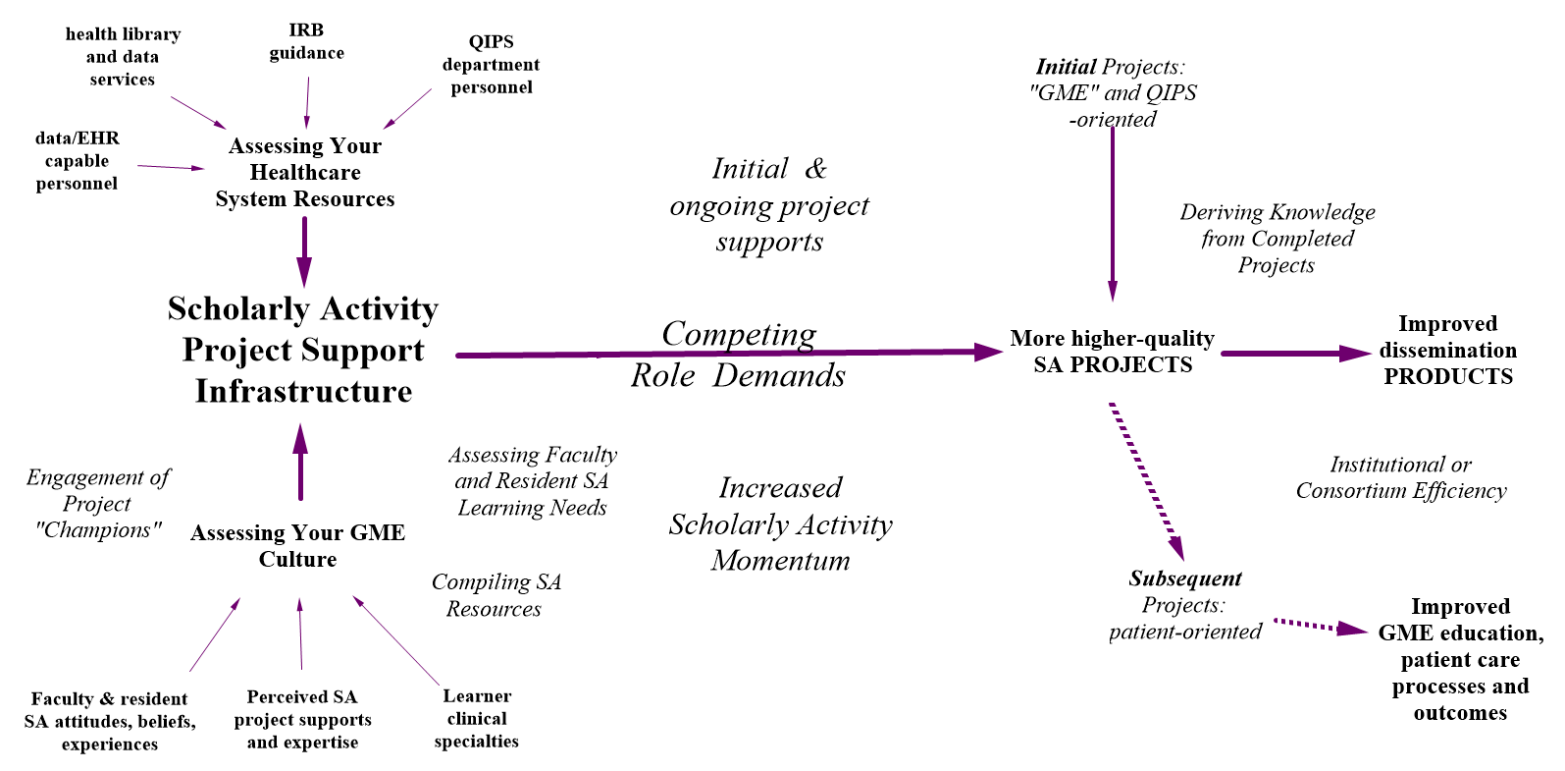
Figure 1 The Statewide Campus System Scholarly Activity Planning Framework for Community-based GME Leaders

Referring to the upper-left of Figure 1, an effective SA support infrastructure should be oriented toward first developing projects that specifically capitalize on available specific resources, such as institutional review board (IRB) guidance, health library and data-capable personnel,^61^ and QIPS/department personnel.^41,65,66^ SA support infrastructure should also be compatible with GME *cultural* realities: Faculty and resident SA attitudes and beliefs, varied specialty perspectives, perceived availability of project supports and mentoring.^14,55,64,67–70^

Referring to the middle of Figure 1, certainly each SA project will need to be developed within the context of each project team’s competing role demands (e.g., patient care commitments, committee work, etc.).^5,14,19,25,34,50^ Ideally, any initially available project-related resources will be sufficient for earlier SA projects, although subsequent project designs can capitalize on better organized resources.

Referring to the right of Figure 1, over time, both the volume and quality of SA projects and dissemination products (e.g., posters, podium presentations and publications) can be expected to increase and GME leaders may be able to realistically expect that improved SA levels will eventually impact patient care processes and outcomes.^20,30,57,59,71–74^

### Developmental SA Strategies

The following ongoing developmental strategies are encouraged for community-based GME leaders striving to attain an organized SA project support infrastructure:

- **Periodically assess your project planners’ SA learning needs and preferences by promoting available resources in an ongoing manner.**

It is also been shown to be necessary to periodically gauge the key learning needs of faculty and residents concerning SA projects.^4,12,14,19,22,75,76^ This information can be obtained through learner surveys and/or initial project planning discussions reviewing project planning materials with project leaders.^33,41,49^ For example, the authors and others have consistently found that project planners from different clinical specialties (e.g., primary care versus surgical specialties) tend to first prioritize different aspects of project development (e.g., evaluating project feasibility, selection of measures, or data set/analytic preferences).^55,75,77,78^

The SCS authors have generally concluded that many primary care physicians tend to prefer GME or QIPS project designs than surgical/procedure-oriented clinicians more oriented to complex research designs (e.g., randomized controlled trials).^42,75^ If found to be present in a leader’s GME setting, these SA project orientations can be incorporated into project planning materials for different learner groups.^8,75^

GME leaders can certainly improve perceptions of available SA project resources by purposefully disseminating information concerning library, electronic health record and QIPS department materials and resources.^6,11,25,47,48,65,71^ The systematic in-house compilation of project planning materials (e.g., *Plan-Do-Check-Act* planning sheets, pertinent articles, project conduction checklists, etc.) can prove key for first meeting the needs and preferences of lesser experienced project learners.^11,25,47,48,71^

GME officials should also periodically consider how their respective IRB have been distinguishing different types of SA projects by perceived level of research risks or types of suggested IRB applications (i.e., *non-human subjects, exempt, expedited, full review*).^59,64,71^ The authors’ have routinely encouraged project developers to sit down with their system IRB contact person when considering initial project design options.^56^ The suggestions from an IRB contact and even the manner in which IRB application forms are formatted may provide novice project planners with additional guidance.^62,75^

- **Orient your project planners to a targeted set of feasible project topics.**

It may become necessary to limit SA project developers to a focused set of topic areas during their preliminary project planning.^30,36,64^ This step can facilitate: a) faculty project mentoring, b) identification of project topics, and c) help project leaders recruit others to participate.^30,54^ Guiding novices with more feasible project topics can help reserve the use of personnel with specialized project skill resources (e.g., qualitative or quantitative analytic skills, extracting secondary EHR chart data, etc.).^38,39,48,75,79^

It is especially important for SA leaders to consider that GME and QIPS-oriented projects generally involve less *protected health information* and will be more rapidly IRB approved.^2,41,71,80^ These types of projects generally entail less work than projects requiring patient recruitment, consenting and follow up contacts. Although GME and QIPS projects may not be as immediately appealing to novices with grandiose project visions, they may prove easier to monitor for GME faculty and officials.^18,28,41,42,80^

- **Strategically engage current/prospective project “champions.”**

GME officials will often need to politically activate and engage a subset of project *champions* to generate an ongoing *critical mass* of SA momentum.^25,29,35,58,67,75–78,81,82^ Increased physician interest in project participation can be generated from project planning workshops with champions present and overtly promoting successful SA project results in newsletters/announcements.^8,11,72,75^

One training mechanism that the SCS authors have twice used is the American Association of Medical College’s “Teach for Quality” (Te4Q) program.^83^ During the SCS offering of this program offering, a cohort of affiliated GME faculty were mentored over a 13 to 15-month period to design, conduct and disseminate the results of their SA projects.^81^ The purposeful selection of capable faculty for this type of structured training program can be vital since prospective applicants may have varying levels of experience, motivation and within-system support from already busy faculty and residents.^22,35,40^

One exemplar Te4Q learner was an emergency medicine physician who completed the program in 2016 after testing a QIPS curriculum with his senior chief resident.^42^ After the program, he adjusted his curriculum for second-year residents developing QIPS and GME-oriented projects with assigned faculty mentors. Individual projects entailed topics such as surveying residents and faculty concerning aspects of their overall GME experiences, testing the pre-post learner impact of GME workshops, and examining the impact of modified pneumonia order set outcomes.

He had subsequently delivered this curriculum to two cohorts of residents with little SCS support. His curriculum-related projects have subsequently contributed at least a dozen regional/national resident poster presentations and two SA publications to date.^42,84^ At the same time, however, the SCS authors have still been consulted for faculty and resident SA project services in other residency programs within this same system. The authors have confirmed the following dissemination products from 2015-2017 Te4Q participants to date: a) 24 local/regional project posters, b) eight statewide posters, c) nine national posters, d) 14 statewide or national podium presentations, e) one (successful) grant proposal application, and f) one poster conference award.

- **Systematically embed SA planning activities into your pre-existing GME processes/groups.**

The GME literature contains a growing number of examples of how SA experts have incorporated project planning/evaluation activities into their current GME program/staff meetings.^10,42,58,59,69^ Residency program leaders may choose to periodically assign review and discussion of novices’ SA project planning drafts or draft manuscripts during scheduled faculty/resident meetings.^8,42,75,77^

This approach may help SA activities become perceived as a *regular part of doing business* in the minds of more residents and faculty.^18,30,50^ Implementing such sequences into pre-existing GME activities may serve to improve both the quality of IRB applications and submission journal drafts, as well as enable novices to become more accustomed to receiving and exchanging critical feedback.^8,42,77^

- **Conduct a postmortem evaluation of every SA project and attempt to derive pragmatic system-specific knowledge for future projects.**

GME officials may underestimate the impetus for future projects potentially derived from completed SA projects.^13,41,42^ Experiences derived from most SA projects can serve to inform what projects may, or may not, work in a specific GME environment/healthcare system. The authors have sometimes conveyed a *football coach* analogy to GME officials striving to strengthen their SA *team* over several years by evaluating SA project successes and lessons. This routine practice of informing project novices about earlier project successes/failures may be integral for them to develop system-compatible projects. It is also unrealistic to expect a subset of residency programs or personnel to conduct most ongoing SA projects.^11,41,85^ GME leaders may wish to implement some type of *writing mentor* mechanisms since the responsibility of project leaders to report their community-based results in the GME literature remains increasingly important.^8,14,58^

The growing complexities of publishing one’s final project results in many GME journals due to increased article processing fees and greater publication competition certainly needs to be acknowledged.^10,63,75^ GME officials may need to routinely attempt to moderate the expectations of somewhat traumatized project authors after their initial paper submissions are rejected.^4,26,38,75^ The compilation of publicly accessible scholarly writing tip sheets and tutorials may therefore be appreciated since most GME settings continue to lack internal paper editing, formatting and submission resources.

- **Consider how to attain maximal SA efficiencies in your GME environment.**

Since many community-based GME leaders continue to experience worsening resource constraints, it may only be realistic to offer workshop/training content through coordinated asynchronous and online mechanisms.^17,81,85–89^ For example, the first author developed a series of 12-to-22-minute QIPS modules in 2016 for online delivery to statewide affiliated learners using *Desire To Learn* online course management software. ^90^ Module topics included: a) *QIPS in Healthcare: Origins and Principles, b) Research and QIPS: Differences and Similarities, c) Feasible Project Design Strategies, d) Preparing your IRB Application, and e) Building a Program of Scholarly Activity as a GME Leader.* Between January 2016 and March 2018, 87 users have been enrolled and made over 280 group/individual module hits.

A growing number of GME experts have also indicated that it may become increasingly impractical for individual settings or programs to achieve sustainable SA progress without some type of intuitional/consortium coordination.^9,10,62,76,89,91^ The SCS authors have been regularly assured by community-based colleagues that they experience increased pressures to maximize the efficiency of their programs and activities as their respective systems have continued to trim resources based on decreased patient care and GME revenue streams.

## CONCLUSIONS

There will never likely be any standardized solutions for development of sustainable project support SA infrastructures across our nation’s diverse GME settings.

This paper presents a non-sequential developmental planning approach comprised of strategies for community-based GME officials striving to address emerging accreditation standards. As GME setting conditions change, leaders’ implemented SA project supports may require ongoing trial and error adjustments.^25,28,30,39,91^ Ideally, this proposed developmental framework can be applied to help meet the diverse SA needs of our nation’s community-based GME environments.

### Conflict of Interest

The authors declare no conflict of interest.
